# Predicting Defects Using Information Intelligence Process Models in the Software Technology Project

**DOI:** 10.1155/2015/598645

**Published:** 2015-10-01

**Authors:** Manjula Gandhi Selvaraj, Devi Shree Jayabal, Thenmozhi Srinivasan, Palanisamy Balasubramanie

**Affiliations:** ^1^Department of Computer Applications, Coimbatore Institute of Technology, Coimbatore, Tamil Nadu 641 014, India; ^2^Department of Electrical and Electronics Engineering, Coimbatore Institute of Technology, Coimbatore, Tamil Nadu 641 014, India; ^3^Department of Computer Applications, Gnanamani College of Technology, AK Samuthiram, Pachal Post, Namakkal District, Tamil Nadu 637 018, India; ^4^Department of Computer Technology (PG), Kongu Engineering College, Perundurai, Tamil Nadu 638 052, India

## Abstract

A key differentiator in a competitive market place is customer satisfaction. As per Gartner 2012 report, only 75%–80% of IT projects are successful. Customer satisfaction should be considered as a part of business strategy. The associated project parameters should be proactively managed and the project outcome needs to be predicted by a technical manager. There is lot of focus on the end state and on minimizing defect leakage as much as possible. Focus should be on proactively managing and shifting left in the software life cycle engineering model. Identify the problem upfront in the project cycle and do not wait for lessons to be learnt and take reactive steps. This paper gives the practical applicability of using predictive models and illustrates use of these models in a project to predict system testing defects thus helping to reduce residual defects.

## 1. Introduction 

A project is temporary endeavor with defined objectives. Project management involves managing the project throughout the life cycle. Project life cycle includes initiation phase, planning phase, executing phase, monitoring and control phase, and finally closedown phase. The challenge lies in understanding and meeting the project goals with the defined project constraints. Every project is unique and needs to be planned well. A project has a defined start and end date. Project management is applicable across industries like production, information technology, and textile to name a few. In the information technology industry, project management plays a crucial role. Industry experts have highlighted the importance of project management. 20%–25% of IT projects fail due to poor project management. Project management principles need to be understood well by the technical managers. Proactive management is the key for success of any project. Ability to predict project outcomes and take preventive actions will determine the success of the project. The focus on shifting left in the project life cycle is vital. Self et al. [1] highlighted the importance of customer satisfaction measurement. Johnson and Gustafsson [2] and Peppers and Rogers [3] studied how customer satisfaction can be improved and customer relationship managed using strategic frameworks. McConnell and Huba [4] discuss how loyal customers become a volunteer sales force.

Shift left approach is one of the approaches where the focus is to concentrate on the upstream activities. The intent is to reduce the defect leakage upfront such that there is less impact on downstream activities. This approach is applicable for any type of industry. In a software development project, the shift left refers to section of quality management concerned with prevention planning. Designing the shift left strategy is important. Focus should be to improve overall operational efficiency and ensure early defect detection while reducing risks and costs. The process phases for a typical software life cycle project are definition phase, design phase, development phase, test phase, and finally deployment phase. A management layer ensures these processes are followed as planned. Shift left strategy needs to be inbuilt in this process so that the technical manager can identify defects upfront.

Stoddard [5] highlighted the importance of process performance models and its reliability in software projects. Gummeson [6] highlighted the use of qualitative methods in managing research. Breyfogle III [7] discussed implementing six sigma qualitative methods. Venkatesh et al. [8, 9] highlighted the importance of process performance models to improve customer satisfaction. Client objectives should be translated into quantitative objectives. These objectives should then be converted to project goals. For each of the goals based on the contract, the service level agreements should be tabulated. Technical manager can review these goals and go with client goals or if organizational goals are even stringent, manager can set that as the project goal. After the goals are clear, operational definition for each of these goals should be documented and agreed upon. This should also include the metric, measures, collection mechanism, and frequency of collection. Kan [10] explains the importance of measures and metrics. Remenyi et al. [11] studied the effective measurement of IT costs and their benefits. As part of measurement, process and product measures need to be considered. Influencing factors for these measures should then be identified. Based on the measures and influencing factors, predictive models can be built to predict project outcomes. Coghlan and Brannick [12] explained the importance of research analysis in the organization using project outcomes. CMMI [13, 14] and Capability Maturity Model explain in detail about the capability models. Kulpa and Johnson [15, 16] explain the importance of CMMI as a process improvement approach. Prediction models can be used to predict interim and final outcomes. Influencing factors can then be modified to analyze the impact and determine actions to be taken. Stutzke [17] highlighted the importance of estimation in software intensive systems.

## 2. Defect Prediction Model

System testing is an important phase in project development life cycle. At this phase, systems are tested extensively. This also includes the integration of systems. For a technical manager, overall testing defect density is an important parameter to track. The number of defects identified during system testing determines the quality of the development. Project subject matter experts identify the different parameters that influence the system testing defect density through brainstorming session. Based on the influencing factors, the key ones that impact the system testing defect density are chosen. Then the operation definition, metrics, and measures are arrived at. Project data was collected for these parameters. Linear regression was performed against the data to find out the key variables that influence the overall testing defect density. After many trial and error methods the two variables below were established as the *X* factors.

With normal data, collected for outcome and the contributing factors, regression equation can be arrived at. Referring to the theory behind regression equations, it can be well understood that the equation depicts the most accurate mode. The fundamentals of regression equation aim at minimizing the sum of squared errors to zero:

(1) dsseda0,dssedb=0,dssedc=0,

where sse is the sum of squared error

(2) $$\begin{array}{c} {\left[\;y_{i}-\left(\;ax_{1}+bx_{2}+c\;\right)\;\right]}^{2}=0. \end{array}$$

(1) *Y* is overall testing defect density—number of defects identified in system testing of the project against effort spent during testing phase of the project.
(2) *X*1 is technology experience—average relevant technology experience of the team, in person months.
(3) *X*2 is design review issue by size—number of design issues identified in the design phase of the project against the size of requirements measured in function points.

Correlation matrix between the variables is determined using *R* [18], a statistical tool.

## 3. Defect Density Regression Equation

The project data collated for the *X* and *Y* factors are as shown in Table 1. Data points from 25 projects in an organization were collected and considered for analysis. The nature of projects selected was large scale IT projects developed under Microsoft C# platform. Attributes such as number of lines of code written, number of defects, and number of human hours spent in the project were collected. The types of defects are lack of domain knowledge, improper algorithm, ambiguity in requirement documentation, incorrect usage of design tool, limitations of GUI controls, and so forth. Defect density is defined as follows:

(3) $$\begin{array}{c} \text{Defect Density }\left(\;\text{DD}\;\right)=\frac{\text{Number of defects}}{\text{Number of lines of code}}. \end{array}$$

**Figure 1:**
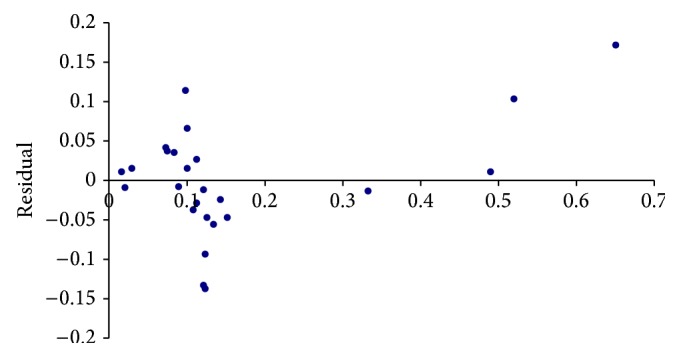
Residual plot.

**Figure 2:**
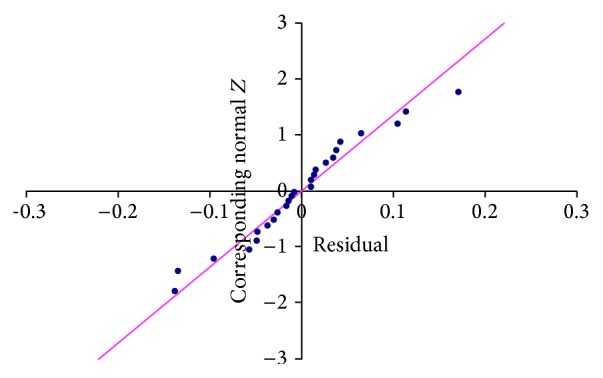
Normal probability plot.

The project data collated for the *X* and *Y* factors are as shown in Table 1. Data points from 25 projects in an organization were collected and considered for analysis. Projects factored in were similar in nature. The null hypothesis considered is that *X*1 and *X*2 have no influence over *Y* (i.e., technology experience and design review issues by size do not impact system testing defect density). No mirror pattern can be found in Figure 1 and hence no heteroscedasticity is found. The normal probability plot as shown in Figure 2 is approximately linear. From the figure it is clear that the normality assumption for the errors has not been violated. With regard to the *p* value, since it is 0.0001 (<0.05), the null hypothesis is not valid, which means the variables selected have an impact on overall defect density.

As shown in Table 2, technology experience has a negative influence on overall defect density. As the team's technology experience increases, there are less project defects and hence the overall testing defect density is reduced. The influence of design review issues by size is negative. This means that when the values of design review issues by size are high the overall defect density will be low and vice versa. The more design issues are resolved, the lesser the probability of defect injection is.

The data proves that the overall testing defect density is influenced by design subprocess and technology experience. Project quality group in the organization shares the baseline data for these variables. For each subprocess, based on the type of project, the organization values can be considered. The technical manager can then use these reference values to determine the upper specification limit and lower specification limit for the project. These values will be available for each subprocess parameter. The technical manager can determine which subprocess he would like to control and select the threshold values based on that. For example, in design process, high level design subprocess, organization value might recommend 92% as the baseline value. Mishra et al. [19] explained the process to establish the process performance baseline. Technical manager might decide to refer to this and make it more stringent and use 95% as the baseline value. Based on the current project context, the other subprocess parameters and technology experience values can be considered for prediction.

Based on the selected threshold values, what-if analysis is performed. Going by the process subparameters and their values, overall testing defect density is predicted. The predicted outcome is then compared with the thresholds. Based on the gap, subprocess parameters are further tweaked to understand the variation. It is important to note that while changing the parameters, the technical manager should understand the practical implication in the project. It is not only about the mathematical model, but also about how it can be put in practice. For example, if, based on the prediction model, the team average technology experience is expected to be around 60 months, then the technical manager has to look at the project constraints. So, though the prediction model considers the key influencing factors to predict the overall defect density, the influencing factor values might have an impact on project schedule and project cost, which need to be analyzed.

Technical manager might decide to adjust the predicted outcome based on the project constraints. At every step, the manager should document the assumptions, risks, and mitigation plans. Detailed defect prevention plan should be in place. At every step, the defect, its type, cause, preventive action, owner, and target date should be documented. Thus at every step the quality gates are important. Quality gates need to be defined at every phase in the project life cycle. The quality gates need to be reviewed and approved by the technical manager. Process quality manager should also review these quality gates and suggest improvements if need be. Different root-cause analysis techniques like 5 whys can be used to pin down the root cause. After identifying the root cause the next steps in terms of corrective and preventive actions should also be thought through. It is recommended to have process quality experts review these plans so that they can bring in their experience and highlight any improvements in these plans.

As shown in Table 4, *b* is the coefficient and gives the least squares estimates. *s*(*b*) gives the standard errors of least square estimates for the *x*-variables. *t* gives the computed *t*-statistic. This is the coefficient divided by the standard error. *p* value gives the *p* value for test of hypothesis. Variance inflation factor (VIF) quantifies the severity of multicollinearity in an ordinary least squares regression analysis. VIF for the given data is 6.43.

In requirements phase of project life cycle, shift left approach can be implemented. Shift left testing is a powerful and well-known trend within the software testing community that is largely intended to better integrate testing into the system/software engineering and thereby uncover defects earlier when they are easier and less expensive to fix. There are four basic ways to shift testing earlier in the life cycle, they are traditional shift left, the incremental shift left, Agile/DevOps shift left, and model-based shift left. The shift left model accelerates the attention to quality from the inception of the project, which increases the ability to discover and correct defects when they occur.

In requirement phase, few process steps that can be added are requirement harvesting, requirements review by the right stakeholders, and requirements testability. Requirement harvesting is about not only getting the requirements from the end user and setting them at baseline, but also diving deep into the requirements. Requirements should be explored and understood in detail to understand the business context and objectives clearly. Requirement review is a process that is crucial and it is important to bring the right stakeholders from the business and user community to validate the requirements. From the given requirements it is important to break them down to testable requirements. Of these requirements, identify those which can be tested individually or as a group or those that cannot be tested. This phase is the first step and it is crucial to do it right. The focus is to detect defects early in the life cycle. Test driven development plan should be the focus in design phase. New project development models talk about test driven development. Test cases are written, executed, and based on the failure of the test cases; codes are developed and tested again. Smoke testing and test environment validation need to be included before testing starts. Root-cause analysis at every stage is vital to look at the corrective and preventive actions. These actions need to be implemented to avoid defect leakage and reoccurrence of defects. Technical managers need to be aware of these process improvement activities and implement them in the project. Process quality consultants play a vital role in training the technical managers with relevant case studies so that they can implement these improvement process steps.

As shown in Table 3, SS is sum of squares due to regression and it is a measure of the total variation in *y* that can be explained by the regression with the *x*-variable. df is the degrees of freedom. MS is the mean square; it is a measure of sum of squares divided by the degrees of freedom. Mean square of regression (MSR) and mean square of error (MSE) are the two variables that define *F*.

Consider *F* = MSR/MSE. The *F*-statistic is used to test whether the *y*-variable and *x*-variable are related.

For the given data mean square of regression is 0.247 and mean square of error is 0.0055. *F*-statistic is 45 and the *p* value is zero. This provides existence of a linear relationship between overall testing defect density and the two-variable technology experience and design review issue. *R* ^2^ is a statistic that will give information about goodness of fit of a model. In regression equation, *R* ^2^ coefficient of determination is a statistical measure to determine how well regression line approximates to real data points. The adjusted *R* ^2^ is a modified version that adjusts the number of predictors in the model. For the given data, *R* ^2^ is 0.8309 and adjusted *R* ^2^ is 0.7861.

Prediction models should help the technical manager to predict the project outcomes. Project outcomes include schedule, costs, and defect parameters. A technical manager has to consider the project requirement, project context, and project constraints, to manage the project successfully. The most excellent approach to defects is to eliminate them as found. This can be only possible if defect prevention techniques and processes are used by the organization. The goal of defect prevention is to eliminate the defects altogether so that they cannot reoccur in the future. The primary objective of proactive defect management is to discover and resolve known defects as early as possible before the occurrence of any major problem related to them. Once the defects are discovered, try to eliminate every defect. For defects that cannot be eliminated try to reduce their impact. The reactive defect management process focuses on identifying the causes of underlying reported problem. A technical manager needs to be proactive in tracking project goals instead of being reactive.

Prediction models are statistical and simulative in nature. These models should help in simulating project scenarios and help in determining outcomes. It can model the different variation factors. These models help the manager with the predicted range or the variation of its outcomes. Based on the different project scenarios, the technical manager needs to perform “what-if” analysis. This analysis will help the manager to change the project parameters based on different scenarios and select the best option.

## 4. Building Quality Gates in Project Life Cycle

A typical software project development life cycle will go through requirements phase, design phase, development phase, unit testing phase, system testing phase, user acceptance testing phase, and finally implementation phase. Quality is an important project parameter at every phase in the life cycle. In the requirement phase, the technical manager needs to ensure that the scope of the requirements is thoroughly documented and signed off. Requirement traceability matrix needs to be created. Every requirement should be traceable as the project moves from one phase to the next phase. Adherence to change management process throughout the process is vital. Technical manager should ensure that change control board is formed during the project initiation phase itself. Requirement clarifications need to be tracked and closed. Requirement reviews should be formally signed off. During the design phase, design prototypes need to be prepared and validated with the user groups.

Design standards, templates, and tools need to be decided and used in the project to ensure compliance. Traceability of design modules to requirements is important. Build sequence needs to be factored as part of design process. The technical manager needs to ensure that coding standards are followed. Configuration management needs to be implemented. Organization configuration tool or client configuration tool can be utilized. Detailed configuration management process should be laid down. The technical manager needs to ensure that all the relevant stakeholders participate in design and code review process. It is recommended to use code review tools. Defect prevention plans need to be implemented.

Detailed test strategy and test plan should be laid down for system testing. Defect management process should be actively followed. Test case coverage is vital. Defect triage meetings are recommended for faster turnaround of defects. Entry and exit criteria at every testing phase should be called out and followed. The technical manager needs to ensure that all business scenarios are covered as part of test coverage. Clearly defined standards and procedures should be implemented. Periodic quality reviews and audits are recommended. The technical manager needs to conduct retrospective meeting and implement the preventive actions. A quality mindset with the project team and all relevant stakeholders is important.

## 5. Conclusion

In any business, customer defines the business. Customer satisfaction is vital for survival. In an IT organization, the customer satisfaction index is pivotal. Customers can make or break business. Customer can in turn act as brand ambassadors and recommend new business for the organization. The main parameters that influence customer satisfaction are faster time to market, quality, and cost. Based on the customer context, the priority of these parameters will change. Few additional parameters can get added. But, overall, cost, quality, and schedule set the base. These three parameters are again related to each other. As a technical manager it is important to understand the customer expectations and ensure that these critical parameters are managed well. Prediction models help in managing these parameters effectively. A manager can predict desired project outcome, perform scenario based analysis, and take right decisions to meet project goals. The practical case study demonstrated how the technical manager can predict overall testing defect density considering technology experience and design review issues by size. The usage of process steps such as prediction model, what-if scenario analysis, corrective and preventive actions, assumptions, and risks helps the manager to meet the project outcomes. Technical managers need to be trained on prediction models and should be comfortable to use these models effectively in projects. Shift left techniques determine the project success. Prediction models are not a one-time activity; it should be continuously used during the project life cycle.

## Figures and Tables

**Table 1:** Project data values.

*Y*	*X*1	*X*2
Overall testing defect density	Technology experience (in months)	Design review issues by size
0.101	32	82.000
0.110	36	72.000
0.090	34	80.000
0.113	33	73.000
0.124	26	55.000
0.123	22	62.000
0.134	23	66.000
0.142	34	69.000
0.125	36	68.000
0.151	19	65.000
0.099	35	90.000
0.020	41	90.000
0.031	43	92.000
0.018	50	93.000
0.073	41	90.000
0.074	43	89.000
0.121	36	74.000
0.113	30	82.000
0.121	24	56.000
0.332	7	43.000
0.520	8	32.000
0.490	9	22.000
0.099	42	97.000
0.085	39	87.000
0.650	11	22.000

**Table 2:** Regression equation.

Intercept	Technology experience (in months)	Design review issues by size
0.6233	−0.0004	−0.0064

**Table 3:** Multiple regression results.

Source	SS	df	MS	*F*	*p*
Regression	0.495	2	0.247	45.099	0.000
Error	0.1207	22	0.0055		
Total	0.6157	24			

**Table 4:** ANOVA table.

	Intercept	Relevant technology experience	Design review index
*b*	0.6233	−0.0004	−0.0064
*s*(*b*)	0.0539	0.0031	0.0018
*t*	11.55	−0.1227	−3.6321
*p*	0.0000	0.9035	0.0015
